# Signatures and prognostic values of related immune targets in tongue cancer

**DOI:** 10.3389/fsurg.2022.952389

**Published:** 2023-01-04

**Authors:** Xiaofei Lv, Xi Yu

**Affiliations:** ^1^ Department of Stomatology, The Second Hospital of Tianjin Medical University, Tianjin, China; ^2^ Department of Anesthesiology, The Second Hospital of Tianjin Medical University, Tianjin, China

**Keywords:** tongue cancer, DEGs, *CCL20*, *SCG5*, *SPP1*

## Abstract

**Background:**

Tongue cancer, as one of the most malignant oral cancers, is highly invasive and has a high risk of recurrence. At present, tongue cancer is not obvious and easy to miss the opportunity for early diagnosis when in the advanced stage. It is important to find markers that can predict the occurrence and progression of tongue cancer.

**Methods:**

Bioinformatics analysis plays an important role in the acquisition of marker genes. GEO and TCGA data are very important public databases. In addition to expression data, the TCGA database also contains corresponding clinical data. In this study, we screened three GEO data sets that met the standard, which included GSE13601, GSE34105, and GSE34106. These data sets were combined using the SVA package to prepare the data for differential expression analysis, and then the limma package was used to set the standard to *p* < 0.05 and |log2 (FC)| ≥ 1.5.

**Results:**

A total of 170 differentially expressed genes (DEGs) were identified. In addition, the DEseq package was used for differential expression analysis using the same criteria for samples in the TCGA database. It ended up with 1,589 DEGs (644 upregulated, 945 downregulated). By merging these two sets of DEGs, 5 common upregulated DEGs (*CCL20*, *SCG5*, *SPP1*, *KRT75*, and *FOLR3*) and 15 common downregulated DEGs were obtained.

**Conclusions:**

Further functional analysis of the DEGs showed that *CCL20*, *SCG5*, and *SPP1* are closely related to prognosis and may be a therapeutic target of TSCC.

## Introduction

Head and neck tumors are the fifth most common malignancies in the world, and smoking and alcohol consumption are two of the most common risk factors associated with these lesions (1). Oral cancer accounts for 2.1% of all new cancer cases worldwide, and squamous cell carcinoma is a known malignancy, accounting for more than 90% of all oral cancers (2, 3). Tongue squamous cell carcinoma (TSCC) is one of the most common malignancies diagnosed in the oral cavity and is associated with a poor prognosis due to its high regional recurrence rate and lymphatic metastasis (4). It is reported that there are about 500,000 new cases of tongue cancer every year, the global incidence of tongue cancer is increasing year by year, and the onset of tongue cancer is getting younger and younger (5). Numerous lymphatic vessels of the tongue are abundant in blood circulation, and the tongue is active frequently. These factors promote the early spread of cancer cells to adjacent tissues and organs, such as lymph nodes, the base of the mouth, throat, and neck. Although tongue cancer treatments are improving as technology advances, the 5-year survival rate is still poor (3, 6). TSCC has a lower survival rate than squamous cell carcinoma elsewhere in the mouth. There is currently a lack of TSCC markers, so finding reliable predictive markers is of great interest (7).

## Materials and methods

### Data preparation

The expression matrix was downloaded from Gene Expression Omnibus (GEO, http://www.ncbi.nlm.nih.gov/geo/). To improve the accuracy of the study, we screened three eligible data sets. These data sets included tongue cancer and normal tissues. GSE13601, GSE34105, and GSE34106 contain 57, 78, and 43 samples, respectively. The SVA software package in R language was used to combine the samples of these three data sets to form a combined data set with 178 samples. Then, we downloaded the expression data of head and neck tumors from the TCGA database and screened the tongue squamous cell carcinoma samples, a total of 147 samples, including 140 tumor samples and 7 normal samples.

### Screening and analysis of differentially expressed genes

The limma package and DESeq package in R language were used for differential expression analysis, and the standard cutoff was set to false discovery rate (FDR) < 0.05 and |log2 (FC)| ≥ 1.5 for both GEO and TCGA, respectively (8, 9). The volcano package and heatmap package in R language were used to visualize DEGs. Kyoto Encyclopedia of Genes and Genomes (KEGG) analysis, Gene Ontology (GO) analysis, and Gene Set Enrichment Analysis (GSEA) were performed for DEGs. Metascape website (https://metascape.org/) was used for pathway enrichment analysis of DEGs in TCGA. The *t*-test was used to compare the expression in the two groups, and ANOVA was used to compare that in multiple groups. We further verified the expression of upregulated DEGs using the GEPIA (http://gepia.cancer-pku.cn/).

### Protein interaction network

The interaction between proteins corresponding to each gene was analyzed using String website (https://string-db.org/), and then the results were visualized using CytoScope. Genemania website (http://genemania.org/) was used for functional enrichment analysis of DEGs.

### Identify the hub gene

The DEGs obtained from combined dataset were crossed with those obtained from TCGA, including 5 up-regulated genes and 15 down-regulated genes.

### Survival analysis

Kaplan–Meier Plotter website (https://kmplot.com/) (citation) was used for survival analysis of the screened HUB genes.

## Results

### Differential expression analysis of the GEO merged data set

**Figure 1:**
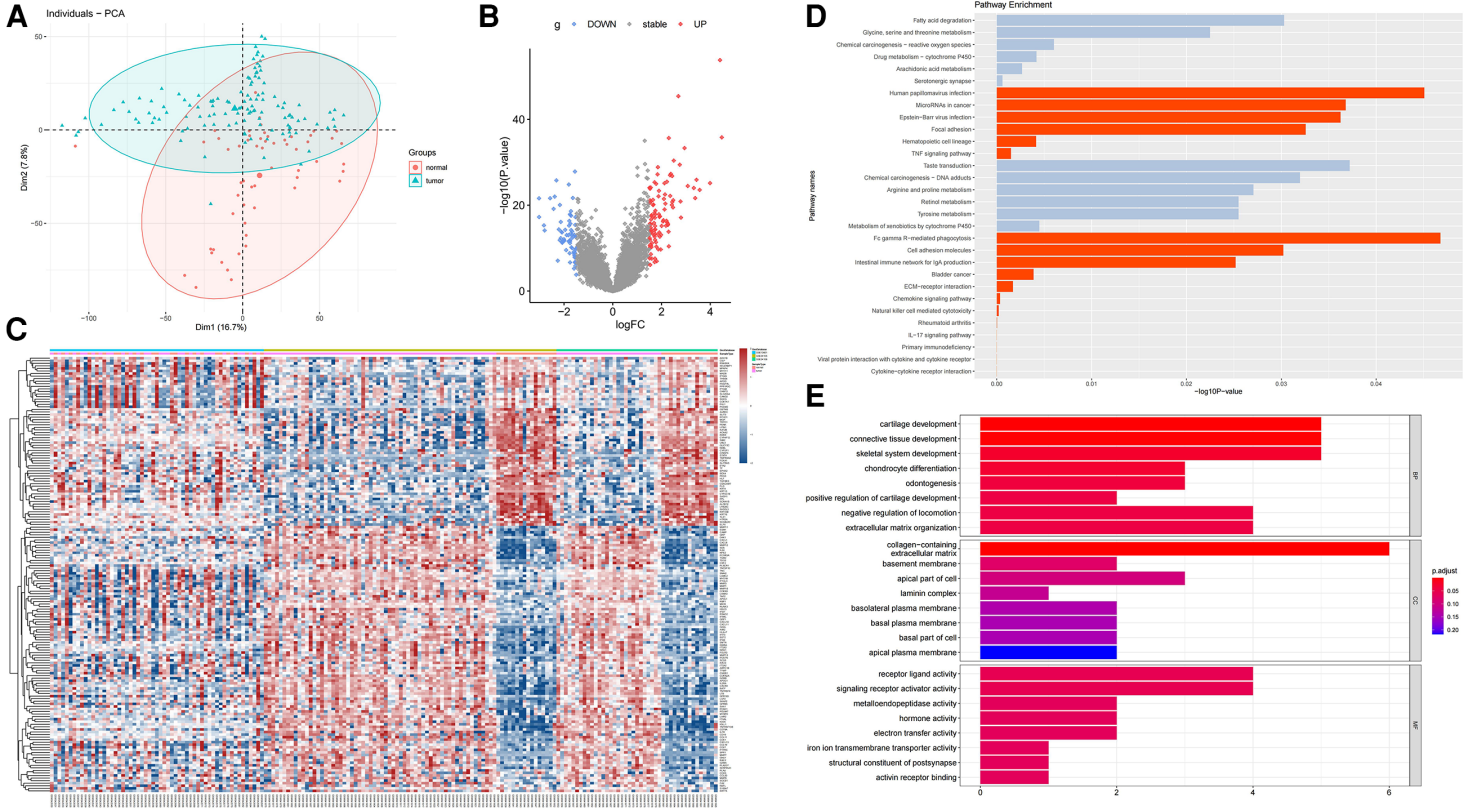
Differential expression analysis of the GEO merged data set (GSE13601, GSE34105 and GSE34106). (**A**) PCA analysis was performed to identify differences between tumor tissue and normal tissue in the combined data set. (**B**) Volcano diagram representing the DEGs obtained by differential expression analysis using the limma package, where red represents upregulated DEGs and blue represents downregulated DEGs. (**C**) Heatmap showing the differentially expressed genes in three data sets, with the annotations above representing each data set and the annotations in the second row representing different types of samples. The differentially expressed genes are shown on the right, where red represents upregulated genes and blue represents downregulated genes. (**D**) KEGG pathway enrichment analysis of DEGs, in which red represents the upregulation gene enrichment pathway and gray represents the downregulation gene enrichment pathway. (**E**) GO enrichment analysis of DEGs.

**Figure 2:**
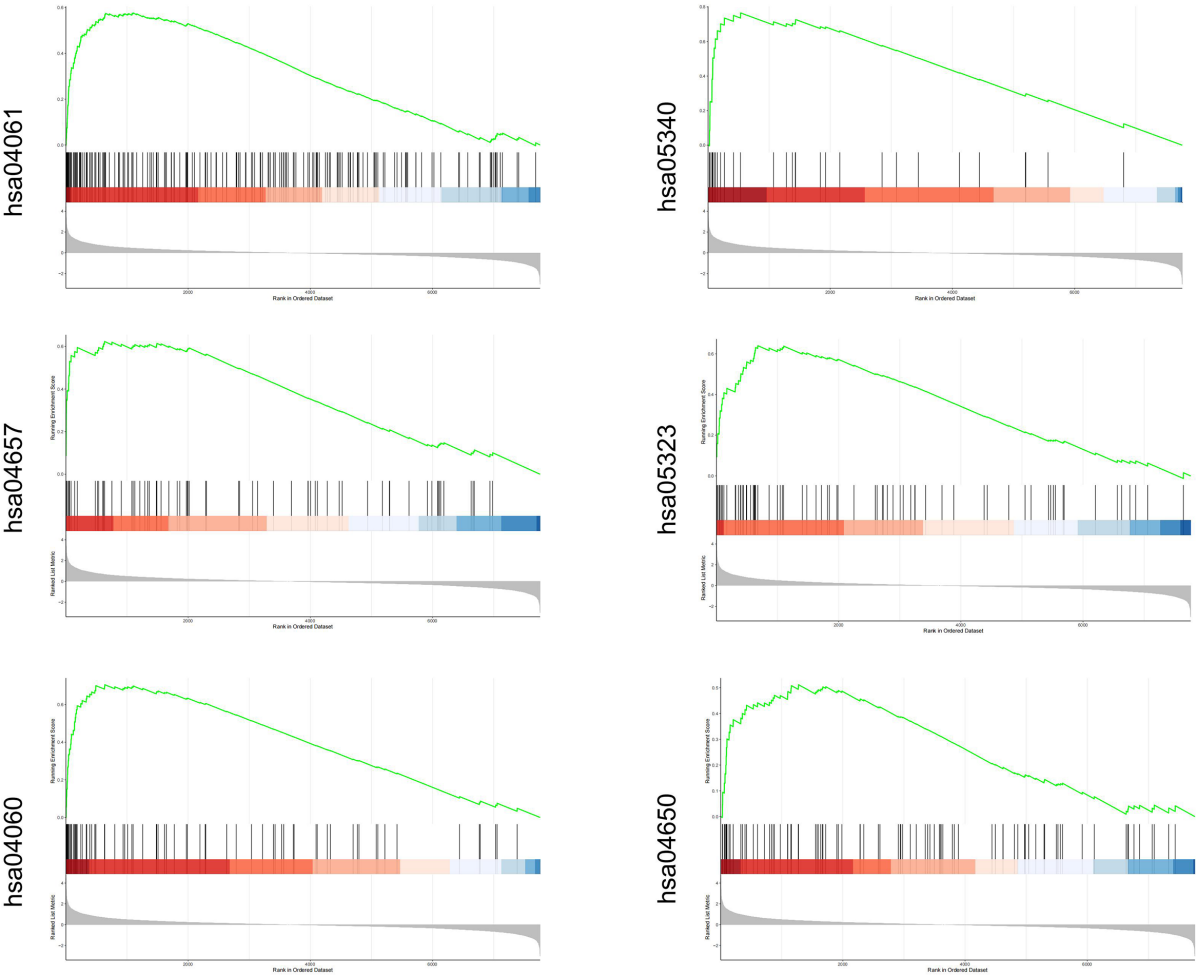
DEG enrichment pathway obtained from the combined database and further verified by GSEA.

We downloaded three data sets GSE13601, GSE34105, and GSE34106 containing tongue squamous cell carcinoma (TSCC) in the GEO database. Each data set contains tumor samples and normal tissue samples. We used the SVA package to merge these data sets and obtain a combined data set. To identify differences between TSCC samples and normal tongue samples, we performed principal component analysis (PCA) and found that the two groups could be separated in the combined data set (Figure 1A). We used the limma package [|log2 (FC)| ≥ 1.5, *p* < 0.05] in R to analyze the 178 samples and screened for DEGs. We identified 170 DEGs, including 104 upregulated genes and 66 downregulated genes, which were shown in the volcano diagram, where the red on the right represents the upregulated differentially expressed genes and the blue one represents the downregulated differentially expressed genes (Figure 1B). In the heatmap, groups were made according to data sets and sample characteristics to explore the expression of different genes. The top annotation represents the different data sets. DEGs are marked on the right side of the figure (Figure 1C). To further explore the functions of the screened DEGs, we used R language for further functional enrichment analysis of these genes in the KEGG database and enriched some up-regulated pathways such as cytokine–cytokine receptor interaction, viral protein interaction with cytokine and cytokine receptor, and primary immunodeficiency (Figure 1D). Then, GO analysis was performed on these genes, and the pathways enriched in upregulated genes were mainly cartilage development, connective tissue development, skeletal system development, and chondrocyte differentiation in the biological process (BP) module (Figure 1E). In the cellular component (CC) module, the enrichment pathways were mainly the collagen-containing extracellular matrix, basement membrane, apical part of the cell, and laminin complex. In the molecular function (MF) module, the pathways were mainly enriched in receptor ligand activity, signaling receptor activator activity, and metalloendopeptidase activity. GSEA analysis was also performed on the first six upregulated pathways, and the results were consistent with the previous analysis (Figure 2). The KEGG correspondence is in Supplementary Table S2.

### Differential expression analysis of TSCC in the TCGA database

**Figure 3:**
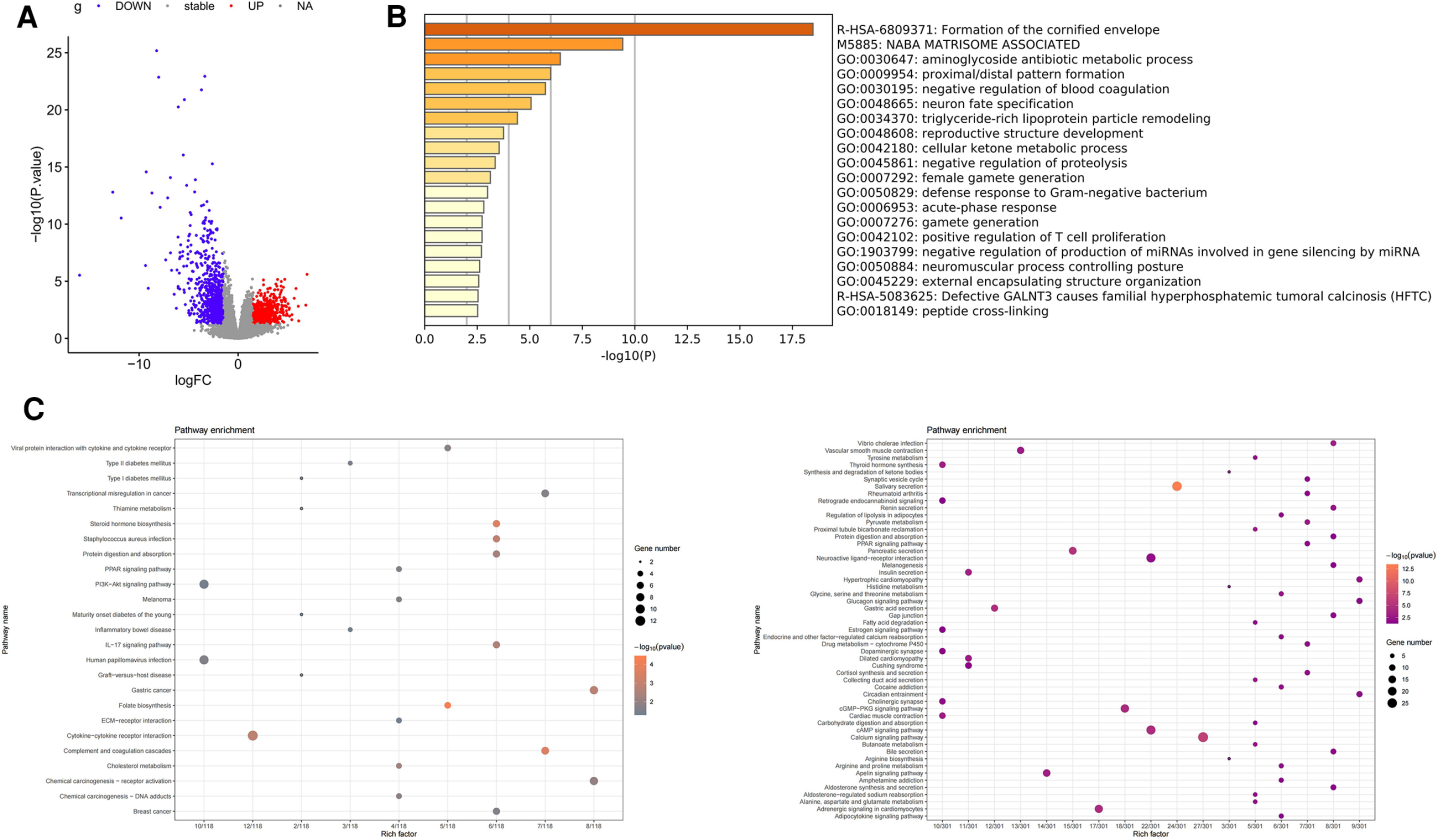
Differential expression analysis of TSCC in the TCGA database. (**A**) Volcano map representing DEGs of TSCC in the TCGA database, with red representing upregulated genes and blue representing downregulated DEGs. (**B**) Results of pathway enrichment analysis of upregulated differentially expressed genes using Metascape. (**C**) Pathway enrichment analysis of DEGs; the left represents the enrichment of the upregulated gene KEGG pathway, and the right represents the enrichment of the downregulated gene KEGG pathway.

DEGs of TSCC vs. normal samples in the TCGA database was performed using the DESeq package in R language [|log2 (FC)| ≥ 1.5, *p* < 0.05]. A total of 644 DEGs upregulated and 945 downregulated DEGs were found, as shown in the volcano diagram (Figure 3A). We used Metascape website to analyze these upregulated DEGs and found that the mainly enriched GO pathways were the aminoglycoside antibiotic metabolic process, proximal/distal pattern formation, and negative regulation of blood coagulation (Figure 3B). KEGG enrichment analysis of upregulated DEGs showed that the upregulated pathways were mainly folate biosynthesis, steroid hormone biosynthesis, complement and coagulation cascades, *Staphylococcus aureus* infection, and cytokine–cytokine receptor interaction. The main downregulated pathways were salivary secretion, calcium signaling pathway, and pancreatic secretion (Figure 3C).

### Acquisition and analysis of hub genes

**Figure 4:**
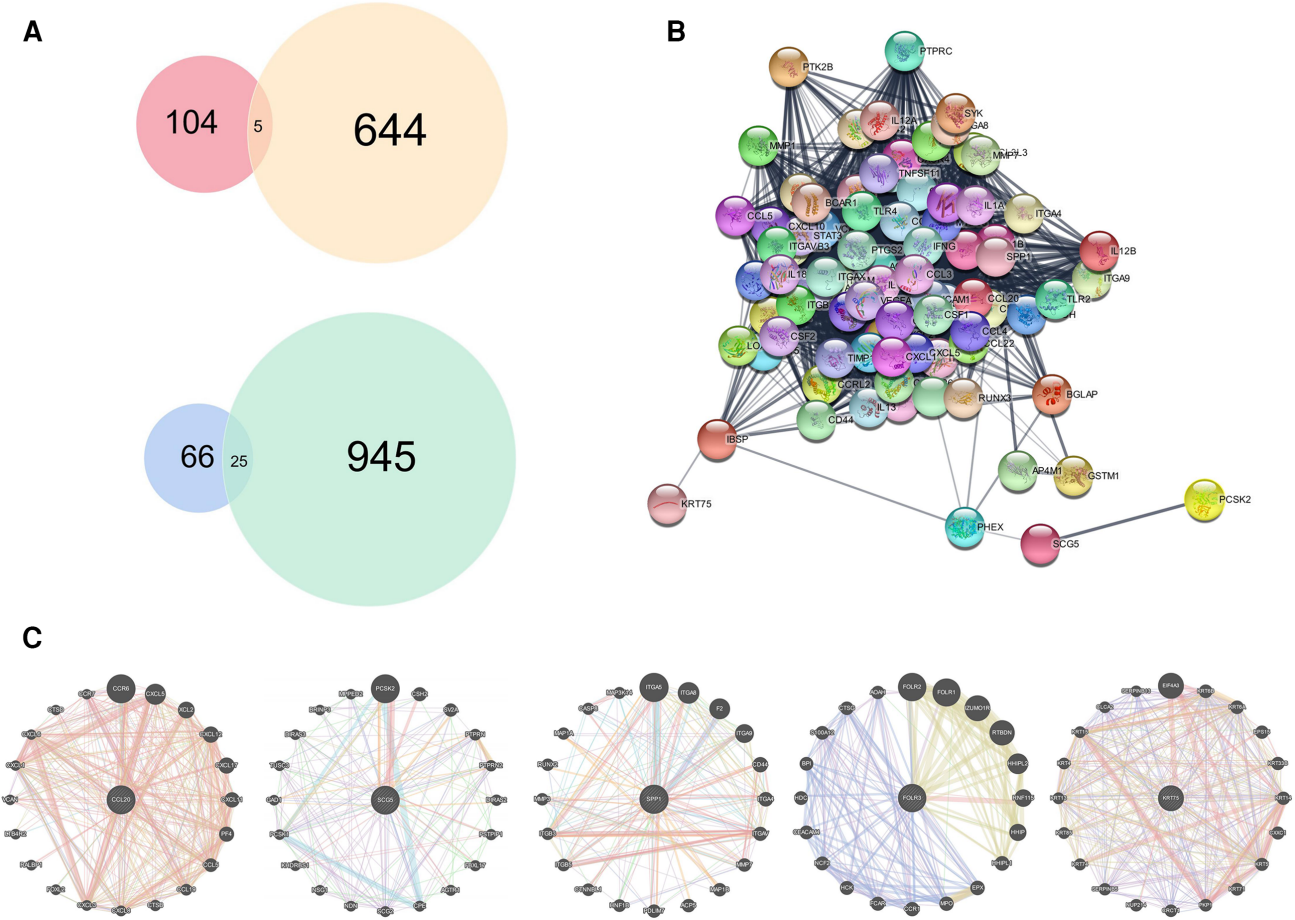
Acquisition and analysis of hub genes. DEGs obtained from GEO merged data set were combined with DEGs obtained from TCGA. (**A**) Venn diagram showing 5 common upregulated DEGs and 15 common downregulated DEGs. (**B**) Protein interaction network analysis of five upregulated genes. (**C**) Functional analyses of the common upregulated DEGs individually.

Differentially expressed genes (DEGs) acquired in GEO were crossed with those in TCGA, and 5 common upregulated genes (*CCL20*, *SCG5*, *SPP1*, *FOLR3*, and *KRT75*) and 15 common downregulated genes (*MAOB*, *HLFSELENBP1*, *CRISP3*, *TMPRSS2*, *TLX1*, *TGFBR3*, *APOD*, *PPP1R3C*, *SLITRK5*, *MFAP4*, *STATH*, *GSTM5*, *RNASE4*, *COX7A1*, *FHL1*, *SLC25A4*, *EFHD1*, *MYH11*, *MGP*, *SCGB2A1*, *LPIN1*, *ELF5*, *ADH1B*, *CKMT2*) were screened (Figure 4A). The protein–protein interaction (PPI) network analysis of these five upregulated genes indicated that proteins expressed by other genes were all functionally related, except *FOLR3* (Figure 4B). Then, we conducted interaction network analysis of these genes individually (Figure 4C).

### Further expression verification of these hub genes

**Figure 5:**
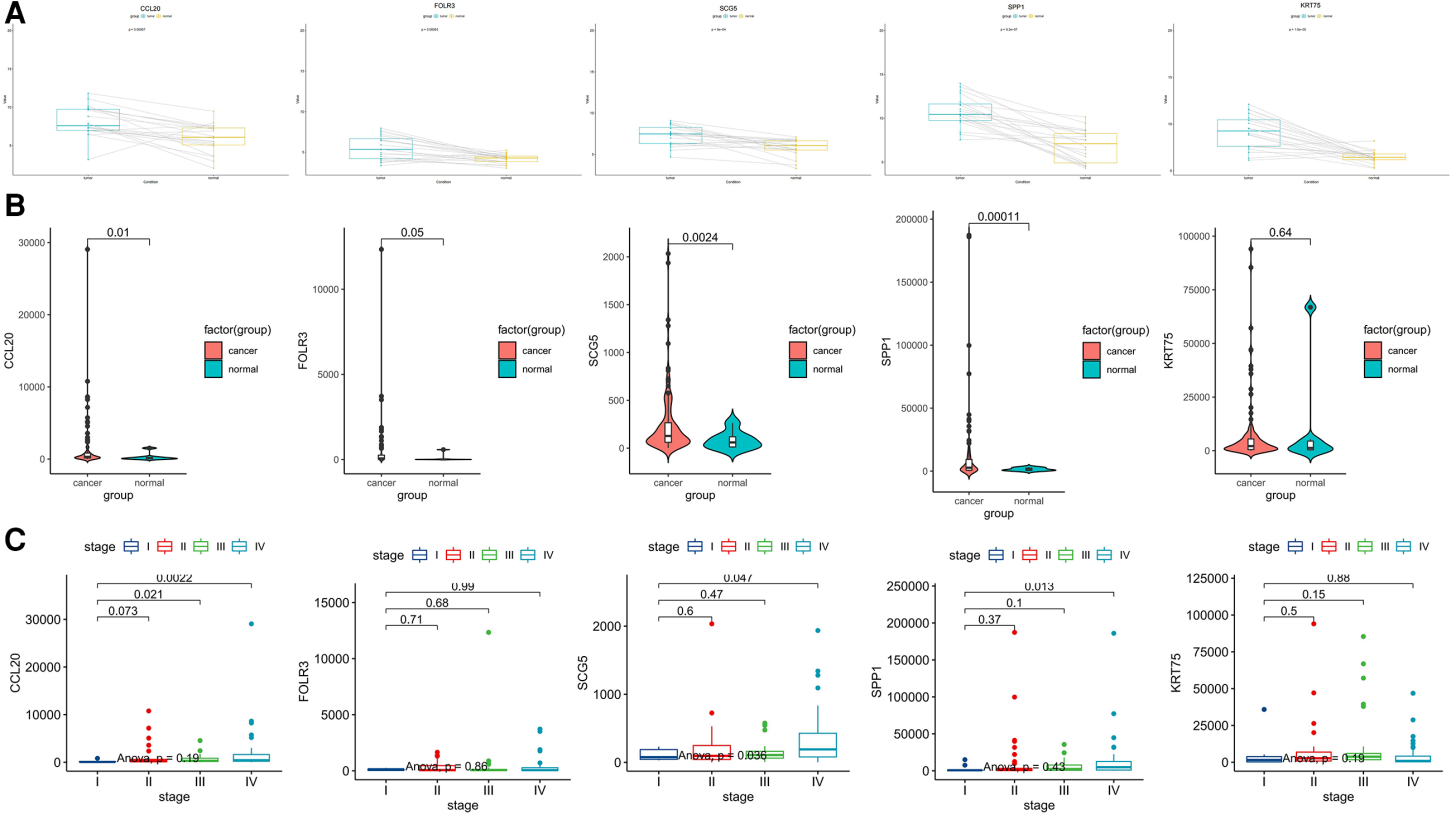
Further expression verification of these five common upregulated genes (*CCL20*, *SCG5*, *SPP1*, *FOLR3*, and *KRT75*). (**A**) Box plots showing the expression levels of these five genes in tumor tissue compared with matched Normal tissue in the GSE13601 database, with lines connected to a pair of paired samples. (**B**) Violin diagram showing the comparison of the expression levels of these five genes in TCGA tongue cancer tissue and Normal tissue. (**C**) Box diagram showing the expression of these five genes at various stages in the TCGA database.

We further verified the expression of upregulated DEGs using the GSE13601 data set. This data set includes tumor samples and their paired normal samples. After removing the individual samples from the database, we obtained 20 pairs of paired samples and then compared the expression levels of *CCL20*, *SCG5*, *SPP1*, *FOLR3*, and *KRT75* genes in tumor tissues vs. normal tissues. It was shown that the expression of these five genes was higher in TSCC than that in normal tissue both in the whole sample and the most paired samples (Figure 5A). The same results can be obtained using the TCGA data set (Figure 5B).

To explore the relationship between the expression of these Hub genes and clinical stage, we used the TCGA database to explore and found that the expression level of CCL20 was significantly increased in patients with stages III and IV compared with stage I (*p* < 0.05) and the expression of SPP1 and SCG5 in stage IV was significantly higher than that in stage I patients (*p* < 0.05) (Figure 5C).

### Multivariate COX regression of TSCC in the TCGA database for each clinical feature and prognosis

**Figure 6:**
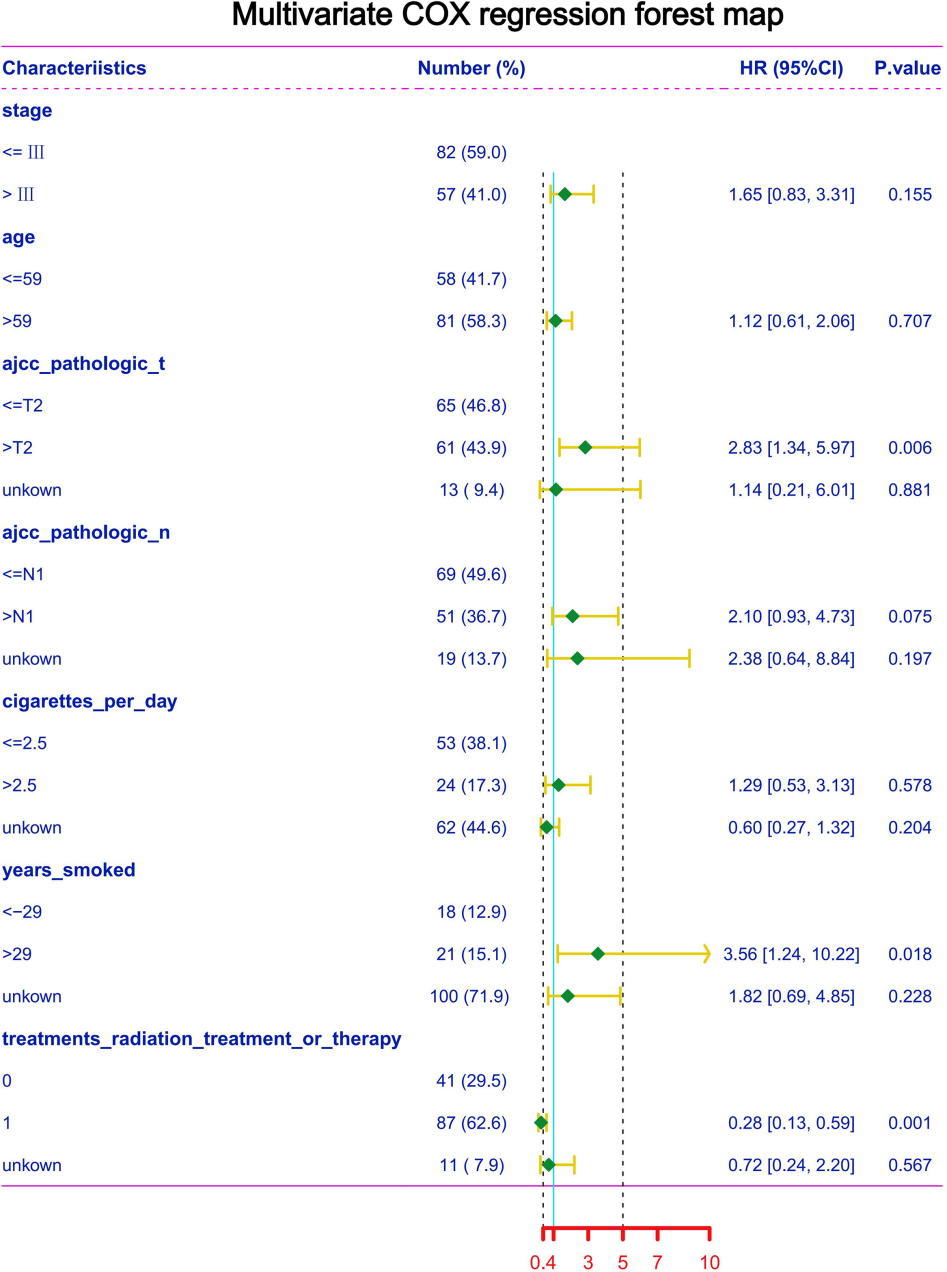
Multivariate COX regression of TSCC in the TCGA database for each clinical feature and prognosis.

**Figure 7:**
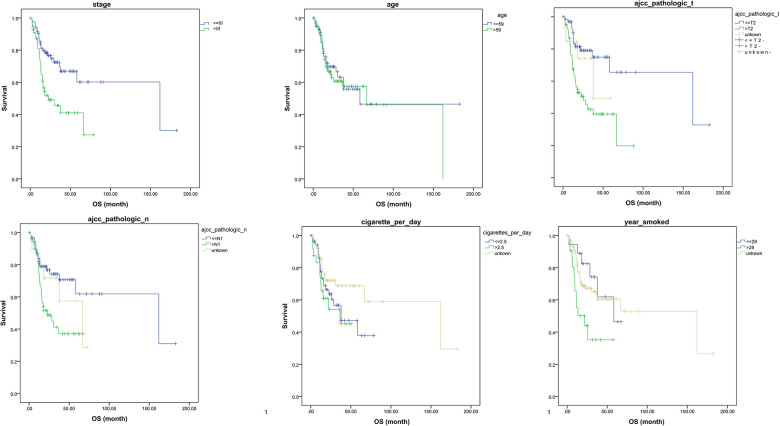
SPSS software used to analyze the individual clinical features and prognosis.

To further explore the clinical data of TCGA, multivariate Cox regression was performed using clinical data from the TCGA database (Figure 6). We find that risk increased when T stage > 2. Similarly, years of smoking also has an impact on the survival of patients; when the smoking time is more than 29 years, the risk is significantly increased. In addition, we found that when the TSCC stage is higher than grade III, age > 59, N stage > 1, and the number of cigarettes smoked per day > 2.5, the risk is increased; however, the *P* value of multivariate Cox analysis did not meet the statistical standards. Kaplan–Meier analysis was performed on clinical data using SPSS software, and we found that patients with a stage higher than III had a significantly worse prognosis than patients with a stage lower than III. AJCC_pathologic_T had a poor prognosis when it was higher than T2. Similarly, the AJCC_pathologic_N stage higher than N1 and smoking time more than 29 years remained risk factors (Figure 7).

### Hub genes analysis in HNSCC

**Figure 8:**
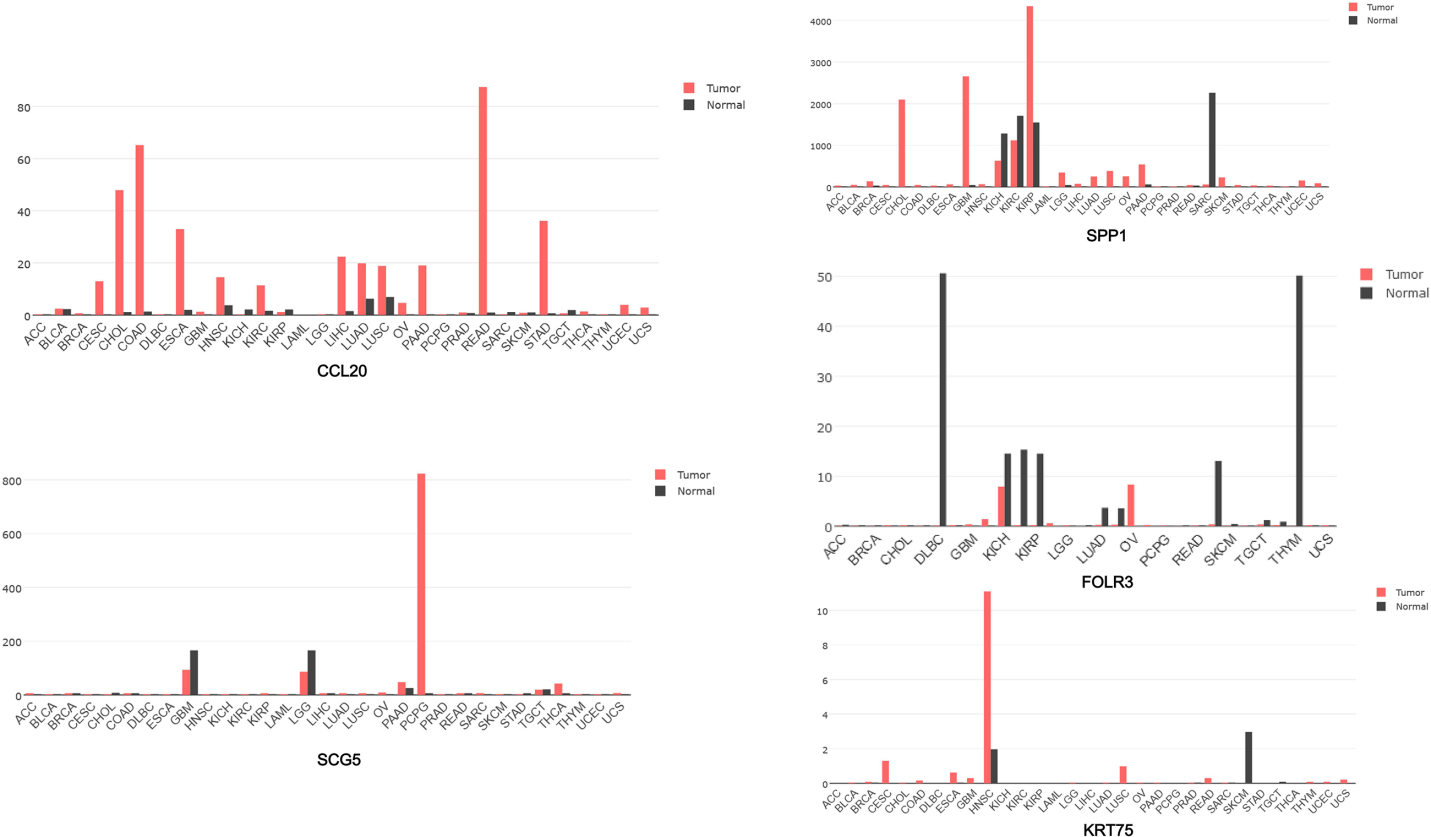
GEPIA software used to analyze the expression of five genes CCL20, SCG5, SPP1, FOLR3, and KRT75 in each cancer species.

**Figure 9:**
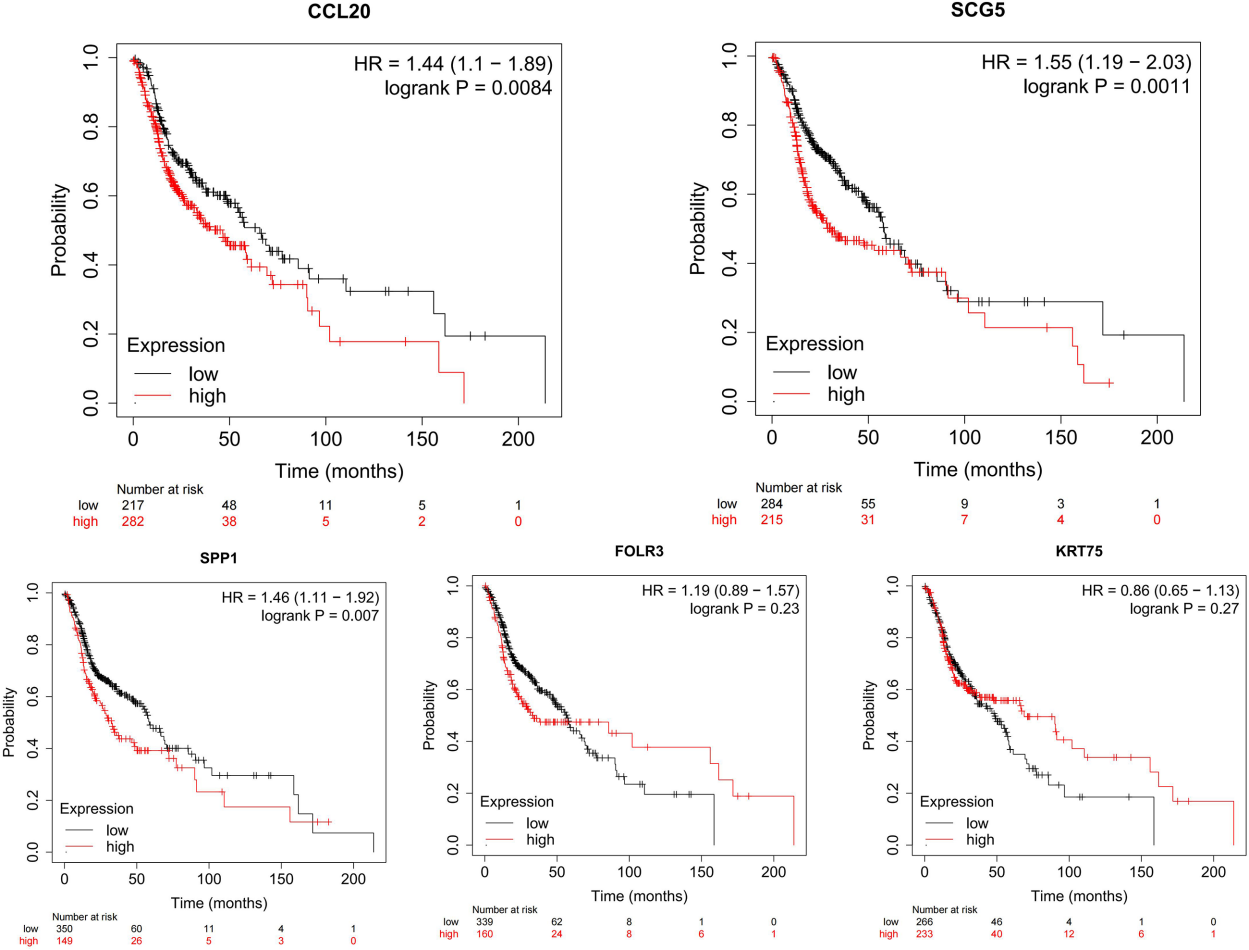
Using Kaplan–Meier Plotter website to identify the relationship between the expression of *CCL20*, *SCG5*, *SPP1*, *FOLR3*, and *KRT75* genes and prognosis in HNCS.

These upregulated hub genes were further verified by GEPIA, and the results showed that the expression of these five upregulated genes in head and neck squamous cell carcinoma (HNSCC) was also higher than that in normal tissues (Figure 8). Kaplan–Meier Plotter website was used to analyze the relationship between the expression of these genes and prognosis, and the results showed that high expression of these genes was strongly associated with poor prognosis in HNSCC (*p* < 0.05) (Figure 9).

## Conclusion

Key molecular alterations need to be confirmed to identify effective therapeutic targets (10–14). To explore the genes related to the occurrence and progression of tongue cancer, we used GEO and TCGA databases for screening. By increasing the sample size, we used the SVA package in R language for GSE13601, GSE34105, and GSE34106. The three data sets were combined to obtain a data set containing 178 samples (121 TSCC and 57 normal samples). By using the limma package for DEG analysis (|log_2_FC| > 1.5, *p* < 0.05), 104 upregulated genes and 66 downregulated genes were screened. Then, the DEGs were obtained by using the DEseq package in R language, and the cut-off value was set the same as before. The DEGs obtained from GEO and TCGA data sets were crossed, obtaining 5 upregulated and 15 downregulated DEGs. The upregulated DEGs are *CCL20*, *SCG5*, *SPP1*, *FOLR3*, and *KET75*.

Chemokine (C–C motif) Ligand 20 (CCL20) is a substance involved in tissue validation and homeostasis with a specific receptor C–C chemokine receptor 6 (CCR6) (15). It is expressed in various tissues and immune cells in human body, and the CCL20–CCR6 axis is closely associated with inflammation and infectious diseases. The CCL20–CCR6 axis is closely related to a variety of cancers, which can directly promote the progress of cancer by enhancing the migration and proliferation of cancer cells and can also regulate the tumor microenvironment by immune cells (16).

CCL20 has been reported to indirectly promote tumor progression by recruiting Treg, Th17, and Th22 cells to maintain the development and immunosuppressive microenvironment (16). Studies have shown that TSCC cells produce CCL20 after interacting with macrophages, and the cells can express CCR6 in the TSCC microenvironment. The CCL20–CCR6 axis may be associated with OSCC progression by inducing CD163 expression in macrophages (17). SPP1 is secreted glycophosphoprotein, which is involved in a variety of functions, including cell adhesion, migration and invasion (18–21). *FOLR3* is highly overexpressed on several tumor cells, including ovarian, nonsmall cell lung, kidney, brain, endometrial, colorectal, breast, pancreatic, gastric, prostate, testicular, and bladder cancer; thus, it is known as therapeutic target for cancer treatment (22–24). It has been reported that the genes are correlated with the progression of a variety of tumors. In addition, according to the results of survival analysis, we found that *CCL20*, *SPP1*, and *SCG5* are closely related to the prognosis of TSCC and HCSN. We have reason to believe that these genes can be developed into prognostic indicators and are expected to be therapeutic targets of TSCC.

## Data Availability

The original contributions presented in the study are included in the article/**Supplementary Material**, further inquiries can be directed to the corresponding author/s.
